# A Neural Model of Auditory Space Compatible with Human Perception under Simulated Echoic Conditions

**DOI:** 10.1371/journal.pone.0137900

**Published:** 2015-09-10

**Authors:** Brian S. Nelson, Jeff M. Donovan, Terry T. Takahashi

**Affiliations:** Institute of Neuroscience, University of Oregon, Eugene, Oregon, United States of America; Harvard Medical School/Massachusetts General Hospital, UNITED STATES

## Abstract

In a typical auditory scene, sounds from different sources and reflective surfaces summate in the ears, causing spatial cues to fluctuate. Prevailing hypotheses of how spatial locations may be encoded and represented across auditory neurons generally disregard these fluctuations and must therefore invoke additional mechanisms for detecting and representing them. Here, we consider a different hypothesis in which spatial perception corresponds to an intermediate or sub-maximal firing probability across spatially selective neurons within each hemisphere. The precedence or Haas effect presents an ideal opportunity for examining this hypothesis, since the temporal superposition of an acoustical reflection with sounds arriving directly from a source can cause otherwise stable cues to fluctuate. Our findings suggest that subjects’ experiences may simply reflect the spatial cues that momentarily arise under various acoustical conditions and how these cues are represented. We further suggest that auditory objects may acquire “edges” under conditions when interaural time differences are broadly distributed.

## Introduction

Prevailing hypotheses of how spatial locations may be encoded and represented across auditory neurons [1–5] have generally focused on conditions when frequency-specific binaural cues, such as interaural time and level differences (ITD and ILD), are stable over time (i.e., coherent). Such conditions can be achieved, for instance, by presenting sounds from a single source in an anechoic chamber. Accordingly, such hypotheses have not addressed the fluctuations in spatial cues arising when sounds from multiple sources, and their acoustical reflections, summate in the ears. Additional mechanisms for encoding and perceiving these fluctuations (e.g., [6]) or for detecting small changes in interaural coherence [7, 8] are therefore necessary [4, 5].

Jeffress’ place theory of sound localization [2], for instance, posits that spatially selective neurons responding maximally to specific combinations of spatial cues may constitute a topographic map of auditory space. An auditory “image” that is focal and well circumscribed is therefore expected when interaural coherence is high, whereas a “blurred” image is expected when interaural coherence is low. While one might infer that the spread of this image provides a cue for interaural coherence, it does not explain how the spread is encoded or why humans report a split image near the left and right ears [6, 9], instead of single blurry image near the midline. The inter-hemispheric hypothesis is another model, which proposes that spatial locations may depend upon the relative levels of activities evoked across spatially selective neurons in the left and right hemispheres [1, 3–5]. Like Jeffress’ hypothesis, however, this hypothesis also leaves the issue of fluctuating binaural cues unaddressed [4, 5].

In the present paper, we consider a different hypothesis for how spatial locations may be encoded and represented, in which spatial perception corresponds to an intermediate or ‘sub-maximal’ firing probability across spatially selective neurons [10–13]. Accordingly, a neural model incorporating this hypothesis describes how small changes in interaural coherence may be detected [7, 8]. The model also predicts that listeners will perceive as single auditory event when spatial cues are stable over time (coherent) but perceive two, spatially distinct, events when fluctuations in spatial cues are large enough [6, 9].

**Fig 1:**
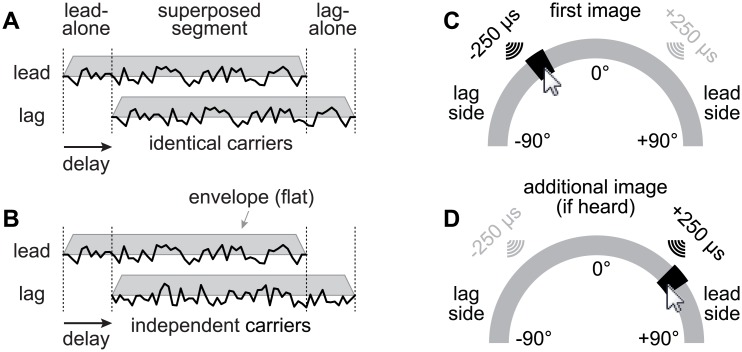
Stimulus segments created by overlapping noise-pairs and the computer interface used in Experiments 1–4. (**A**) Illustration of when the noise-pairs were identical (i.e., correlated). (**B**) Illustration of when the noise-pairs were statistically independent (i.e., uncorrelated, incoherent). The lead stimulus is present alone for a length of time equal to the delay. Both sounds are then present during the superposed segment, as are ongoing delays between corresponding features of the lead and lag stimuli. Finally, the lag stimulus is present alone for the length of the delay. Gray shading shows the flat envelopes that were applied to the noise-pairs. **(C-D)** Listeners were told that each arc represented the frontal hemifield (180°) at eye level. If only one auditory image was perceived, subjects were instructed to indicate the image’s central azimuth on the arc nearest the top of the computer screen **(C)**. If an additional auditory image was perceived, subjects were instructed to indicate its central azimuth on the arc nearest the bottom of the computer screen **(D)**.

The precedence effect or Haas effect [14–16], which is an experimental paradigm for studying spatial hearing under echoic conditions, presents an ideal opportunity for examining this hypothesis. First, the stimuli that are used to study the effect typically consist of a “direct” or “leading” sound that is then followed by a copy of that sound, after a short delay, from another direction (Fig 1A). Such stimuli are ideal for testing our hypothesis because the leading sound is typically present, alone, prior to the onset of the second “lagging” sound. Similarly, the lagging sound is present, alone, after the offset of the leading sound. These two segments, which are referred to as the “lead-alone” and “lag-alone” segments (Fig 1A), are analogous to the “glimpses” that listeners may occasionally be afforded in a typical auditory scene, during which spatial cues are relatively stable (i.e., coherent or correlated) [17–20]. In addition, a third segment is generated, when both stimuli are present, which is referred to as the “superposed” segment. Unlike during the alone segments, the superposition of the leading and lagging stimuli causes binaural cues to fluctuate, as may occur in-between glimpses in a typical auditory scene.

The precedence effect is also well represented in the literature and is characterized by several well-established psychophysical phenomena. When identical sounds arrive simultaneously, listeners perceive a single auditory image located midway between the sources of the two sounds, a condition known as “summing localization”. At short, nonzero delays, listeners report a single sound near the location from which the direct or leading sound was presented. In the literature, the perception of a single image is termed “fusion” in that the two sources appear to be fused into one. The proximity of the fused image to that of the lead is referred to as “localization dominance” [21, 22]. As the delay increases, the simulated reflection or lagging sound becomes perceptible as a spatially distinct event and the listener’s “echo threshold” is crossed. Even when both the leading and lagging sounds are perceived to be spatially distinct, the leading sound may dominate perception in that subjects report it as being more spatially salient than the lagging sound. The lagging sound’s location may also be perceived closer to the lead than its actual location [16, 17, 23]. Thus, localization dominance can persist beyond echo threshold and the location of the lagging sound, when heard as a spatially distinct event, may also be biased toward that of the lead. Finally, the ability to discriminate changes in the lag’s location may diminish at short delays, a phenomenon termed “lag-discrimination suppression” [23].

The first purpose of the present psychophysical study was is to identify contributions to precedence phenomena from the superposed segments of stimuli and to distinguish their contributions from those of the “alone” segments. When the lead- and lag-alone segments are excised from identical noise-pairs (Fig 1A), the leading source continues to dominate spatial perception at short delays [24–27] suggesting that spatial cues in the superposed segment are accessible to the auditory system. Neither source can dominate, however, when the noise-pairs are statistically independent. Contributions to fusion and localization dominance from the alone segments should therefore be evident in the responses to independent noise-pairs (Fig 1B). Conversely, contributions to fusion and localization dominance from the superposed segment should be evident when responses to identical (Fig 1A) and independent noise-pairs (Fig 1B) are compared. In deriving these contributions, we incorporate the ringing of peripheral filters [28–31], which can blur the distinctions between the segments (see S1 Fig), but we make no assumptions about neural interactions between the segments, such as those from lateral inhibition. Responses to identical and independent stimuli were investigated previously by Perrott et al. [32]. To our knowledge, however, our study is the first to attribute differences in the responses to the superposed and alone segments of noise-pairs.

After estimating contributions to precedence phenomena from the various stimulus segments, a neural model is considered to describe how spatial cues arising during the segments may contribute to perception. After passing the stimuli through a bank of auditory filters, the extent to which the envelopes of filtered stimuli may explain localization dominance is examined. The notion that auditory space may be represented by sub-maximal activities across spatially selective neurons is then considered. Empirical observations of fusion and localization dominance are lastly derived from the spatial events that listeners were predicted to have experienced.

## Psychophysical Methods

Experiments were carried out under a protocol approved by the University of Oregon Institutional Review Board for the Protection of Human Subjects. A total of 39 listeners with no self-reported hearing losses, having provided written informed consent, were recruited from the University of Oregon.

Lead and lag stimuli consisted of identical or statistically independent noise-bursts (2.5 ms linear on/off ramps; 0.2–11kHz bandwidth) presented over headphones (Sennheiser HD 280 Pro) with interaural time differences (ITD) of +250 μs (right) or -250 μs (left). Thus, had the lead or lag stimuli been presented separately, intracranial images would have been perceived on opposite sides of the midline, corresponding to the +250 or -250 μs ITD.

Four experiments were performed to explore different stimulus durations and configurations. In Experiments 1 and 2, the stimuli were 200 ms or 30 ms respectively and the lead-lag delay was varied between 0 and 16 ms. In Experiment 3, the stimuli were 10 ms and the longest delay tested was 8 ms, so that the stimuli always overlapped. Finally, Experiment 4 was identical to Experiment 2, except that the lead-alone and lag-alone segments were excised and the stimuli were lengthened, as necessary, to produce a 30-ms long superposed segment. For the independent noise-pairs in Experiment 4, removing the alone segments removes all information regarding the lead-lag delay. Therefore, results from four arbitrary “delays” (0, 0.25, 0.5, 1-ms) were pooled.

Ten subjects were tested in each of the four experiments. Each stimulus was presented 50 (Experiments 1 and 2), 60 (Experiment 3), or 70 (Experiment 4) times in random order, over 5 listening sessions. One subject was tested in both Experiments 3 and 4. The noise tokens differed on each trial, and before each trial, listeners heard a diotic noise burst, equal in length to that of the overall experimental stimulus (10–216 ms) that provided a sense of the midline as a reference.

Stimuli were presented to subjects seated in a noise-attenuating chamber (Industrial Acoustics Co. IAC; 2.2 m × 2.1 m × 2.0 m). Stimulus presentation and data acquisition were controlled using custom software (Matlab, The Mathworks). Sounds were synthesized and presented (48.8 kHz sampling rate) using a real-time audio processor and headphone amplifier (RP2.1, HB6; Tucker-Davis Technologies). The noise bursts, presented alone (i.e., not as part of a lead-lag pair), were approximately 75 dB (re: 20 μPa) at the headphones. The leading stimulus could have an ITD of -250 or +250 μs on any trial. However, locations indicated were analyzed as though the leading stimulus always came from the right (+250 μs ITD) and the lagging stimulus always came from the left (-250 μs ITD) [17].

Listeners were instructed to focus on the “clearest” image heard and to indicate the image’s centroid on an arc representing the possible intracranial positions (-90° [left] to +90° [right]; Fig 1C). If an additional image was heard, they were asked to mark its centroid on a second, lower arc (Fig 1D). This procedure is identical to that used in a previous study [17] and similar to that used by Brown and Stecker [33]. Listeners were given no further instructions regarding clarity to avoid biasing their subjective judgments. The arcs were rendered on a flat computer display, and the perceived loci of the intracranial images were marked on the arcs by the press of a mouse button.

The number of images perceived by the subjects was inferred from the proportion of trials in which an additional image was indicated on the lower arc. The direction and confidence with which the dominant image was lateralized was measured as the average location indicated on the upper arc. When indicated, the direction and confidence with which the additional image was lateralized was determined in the same way from responses on the lower arc. Locations for the individual sources were neither measured nor factored into the analysis because the cues they would have generated (-250 or +250 μs), if presented separately, were seldom observed during the superposed segments of the composite stimuli. In addition, because the auditory images that subjects reported were often spatially indistinct and thus open to interpretation, the locations that subjects indicated most frequently were not directly measured, beyond their contributions to the average locations indicated by the subjects for the various stimuli on the upper and lower arcs.

## Psychophysical Results

**Fig 2:**
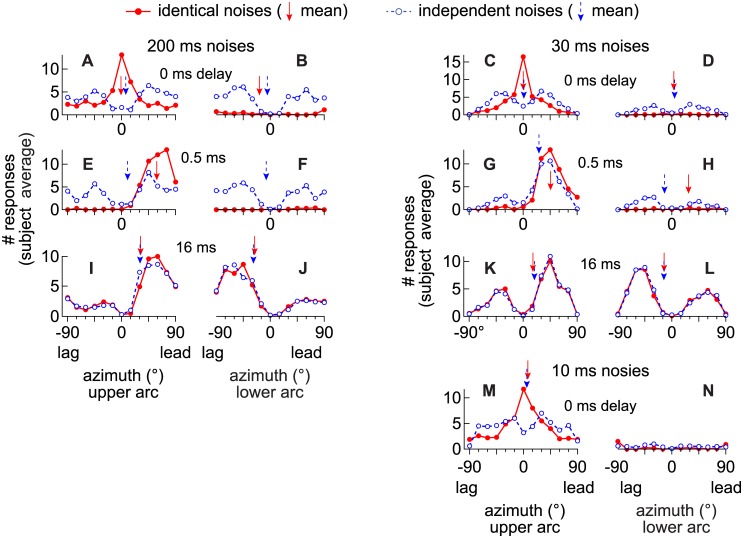
Distributions of angles indicated by the subjects on the top (left distribution) and bottom (right distribution) arcs. Distributions were averaged across subjects and are plotted as if the lead always came from the right (+250 μs ITD) and the lag from the left (-250 μs ITD) [17]. For each distribution, the frontal hemifield is represented on the abscissa with values ranging from -90 (left) to +90 (right). Solid lines and filled circles show the results for the identical noise-pairs. Dashed lines and open circles show the results for the independent noise-pairs. Arrows indicate the means of the individual distributions. **(A- D)** Distributions when there was no delay (0 ms) and the noise-pairs were 200 ms **(A-B)** or 30 ms **(C-D)**. **(E- H)** Distributions when the delay was 0.5 ms and the noise-pairs were 200 ms **(E-F)** or 30 ms **(G-H)**. **(I- L)** Distributions when the delay was 16 ms and the noise-pairs were 200 ms **(I-J)** or 30 ms **(K-L)**. **(M- N)** Distributions when there was no delay (0 ms) and the noise-pairs were 10 ms.

Fig 2 shows the averaged distributions of locations indicated by the subjects (N = 10 per experiment) in Experiment 1 (200 ms; Fig 2A, 2B, 2E, 2F, 2I and 2J), Experiment 2 (30 ms; Fig 2C, 2D, 2G, 2H, 2K and 2L) and Experiment 3 (Fig 2M and 2N). Results for identical (solid lines and filled symbols) and independent noise-pairs (dashed lines and open symbols) at delays of 0, 0.5, and 16 ms (Experiments 1–2) or 0 ms (Experiment 3) are shown.

When identical noise-pairs were presented at a delay of 0-ms (Fig 2A–2D, 2M and 2N, filled circles), the stimuli are diotic and listeners nearly always indicated a single image on the upper arc. Most of the responses were for locations near the midline so the average angle of the distribution, μ, was close to 0° (downward arrows). When the stimuli were independent (open circles in Fig 2A–2D, 2M and 2N), an additional image was indicated on the second arc, especially when the stimuli were 200 ms (Fig 2B). Two modes are therefore evident in the distributions, presumably because neither source was perceived as being “clearer” than the other. Because the distributions are symmetric, μ is still close to 0° (downward, dashed and blue, arrow, Fig 2B).

For identical stimuli at a delay of 0.5 ms (filled circles in Fig 2E–2H), a single image was again indicated in most of the trials, as shown by a higher incidence of responses on the upper arc (E and G) than on the lower (F and H). However, when compared to the 0 ms condition (A-D and M-N), most of the responses were on the leading sides of the arcs (μ > 0°). For the independent stimuli (open circles), shortening the lengths of the stimuli from 200 (Fig 2E and 2F) to 30 ms (G-H) resulted in a higher incidence of responses on the upper arc (G) than the lower (H). Most of the responses were on the leading sides of the upper arcs (μ > 0°; Fig 2E and 2G), whereas relatively few responses were made on the lower arcs (Fig 2F and 2H).

Lastly, Fig 2I–2L show that an additional image was nearly always indicated when the delay was 16 ms, regardless of stimulus similarity. In addition, most of the responses were on the leading side of the first arc (Fig 2I and 2K) but on the lagging side of the lower arc (Fig 2J and 2K), with the bias being stronger for the longer 200-ms stimuli (Fig 2I and 2J).

### Fusion

**Fig 3:**
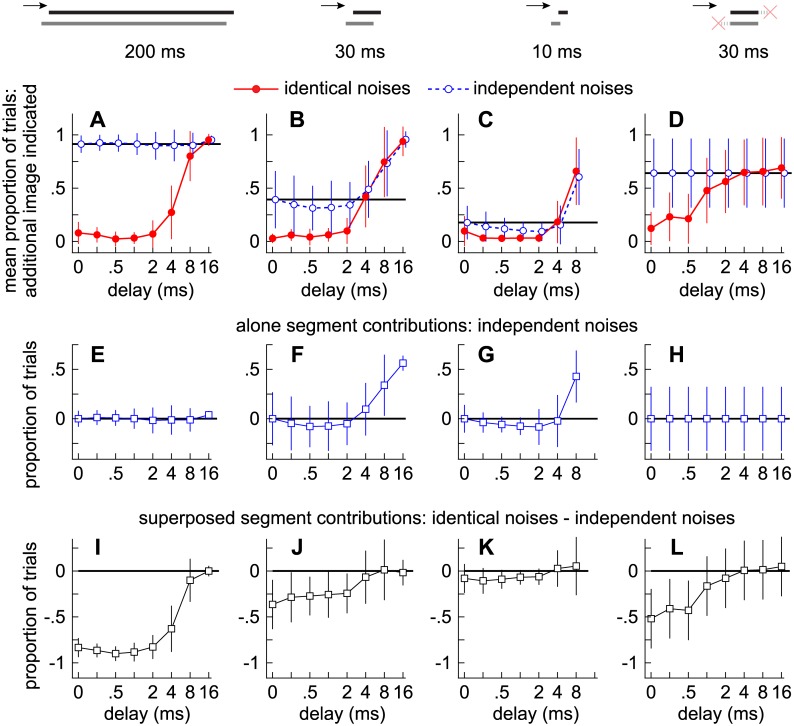
Contributions to fusion from the alone and superposed segments. **(A-D)** Markers indicate the proportions of trials in which subjects indicated a second auditory image at a given delay (abscissa) when the noise-pairs were 200 ms **(A)**, 30 ms **(B)**, 10 ms **(C)**, or 30 ms with synchronized onsets and offsets **(D)**. Error bars indicate variation across subjects (±1 s.d.). Solid lines and filled circles show the results for the identical noise-pairs. Dashed lines and open circles show the results for the independent noise-pairs. **(E-H)** Markers indicate how the proportions differed for the independent noise-pairs relative to when there was no delay and thus the contributions to fusion from the alone segments. **(I-L)** Markers indicate how the proportions differed for the independent and identical noise-pairs and thus the contributions to fusion from the superposed segment.

Fig 3A–3D plot against delay the proportion of trials in which subjects reported an additional image in Experiments 1–4. Since fusion is the tendency to report a single auditory image, a low proportion along the ordinate suggests strong fusion; a high proportion suggests weak fusion. Ten subjects participated in each experiment, and each data point represents the average across the ten subjects (vertical lines show ± 1 standard deviation [s.d.]).

Results obtained with the independent noises (open circles) are first considered, from which contributions to fusion from the lead- and lag-alone segments are inferred. Contributions to fusion from the superposed segments are then inferred by comparing results for the independent (open circles) and identical (filled circles) noises.

#### Contributions to fusion from the alone segments

When independent, 200-ms noise-pairs were presented with no delay (leftmost open circle in Fig 3A), listeners reported two sources in 91% of the trials (± 8% s.d.), indicating very weak fusion. This proportion of trials in which the subjects reported two sources with simultaneous independent noises is hereafter referred to as the “independence proportion” (horizontal line) and reflects the tendencies of the listeners to report two sources in the absence of onset/offset or ongoing cues regarding the lead-lag delay. Note that the listeners consistently report the additional source regardless of delay. Whether the delay is 2 ms or 16 ms, the proportion of trials in which subjects report the additional source barely changes. Deviations from the independence proportion are plotted against delay in Fig 3E.

Results from Experiments 2 and 3, which used shorter stimuli (30 or 10 ms), are shown in Fig 3B and 3C. Compared to the results obtained with 200 ms noise-pairs, the independence proportion (solid line) is considerably lower with the shorter sounds. In other words, as the stimuli were shortened, subjects were less likely to report an additional source. At delays between 0.25 ms and 2 ms, the psychometric functions are lower than the independence proportions (0.39 ± 0.27 and 0.14 ± 0.14, respectively), suggesting that fusion was strengthened by the presence of a short lead-alone segment. The psychometric functions then rise above the independence proportions (Fig 3F and 3G) at delays greater than approximately 2 and 4 ms, respectively, suggesting that fusion is weakened by the presence of a sufficiently long lag-alone segment [17, 20].

#### Contributions to fusion from the superposed segment

Contributions to fusion from the superposed segments of the various stimuli (Experiments 1–4) were deduced by comparing the psychometric functions obtained with identical (filled circles) and independent (open circles) noises (Fig 3A–3D). As shown in Fig 3A, when the noises were 200 ms, the functions obtained for the identical noises (filled circles) are well below the independence proportion for delays between 0 and 4 ms, suggesting that fusion is quite strong. This is also apparent in Fig 3I, which plots against delay the difference between the psychometric functions obtained with the independent and identical noises.

For the shorter noise-pairs used in Experiments 2 and 3, the psychometric functions obtained for the identical noises are similar (filled circles in Fig 3B and 3C) to results obtained with 200 ms the noises (filled circles in Fig 3A). Yet, differences between results obtained with the identical and independent noises, which corresponds to the area beneath the horizontal line and the psychometric functions in Fig 3I–3K, are smaller for the shorter noise-pairs, suggesting that the superposed segment contributed more to fusion when it was longer. In Experiment 4, listeners heard 30 ms noise-pairs with synchronous onsets and offsets (Fig 3D). The differences between results obtained with the identical and independent noises (Fig 3L) are similar to differences observed when the alone segments were intact (Fig 3J), although inter-subject variation was higher when the alone segments were excised. These results suggest that fusion can strengthen at short delays (< ~4 ms) even when the alone segments are excised and cannot contribute to the phenomenon (Fig 3H).

Stimulus similarity was found to have a comparable influence in a previous study by Perrott et al. [32]. Using statistically independent (uncorrelated) 50-ms noise-pairs, an independence proportion of ~0.8 was reported when there was no delay. The proportions then increased for delays longer than 2 ms (i.e., proportions of trials in which subjects failed to report a single source). In comparison to the independent noises, the proportions were substantially lower when identical noise-pairs were tested, rising from 0.3 when there was no delay to 0.63 at a delay of 4 ms and 0.9 at a delay of 10 ms. As in the present study, these results suggest that the superposed segment can cause considerable fusion when the delays between corresponding envelope or carrier features are short.

### Localization dominance

**Fig 4:**
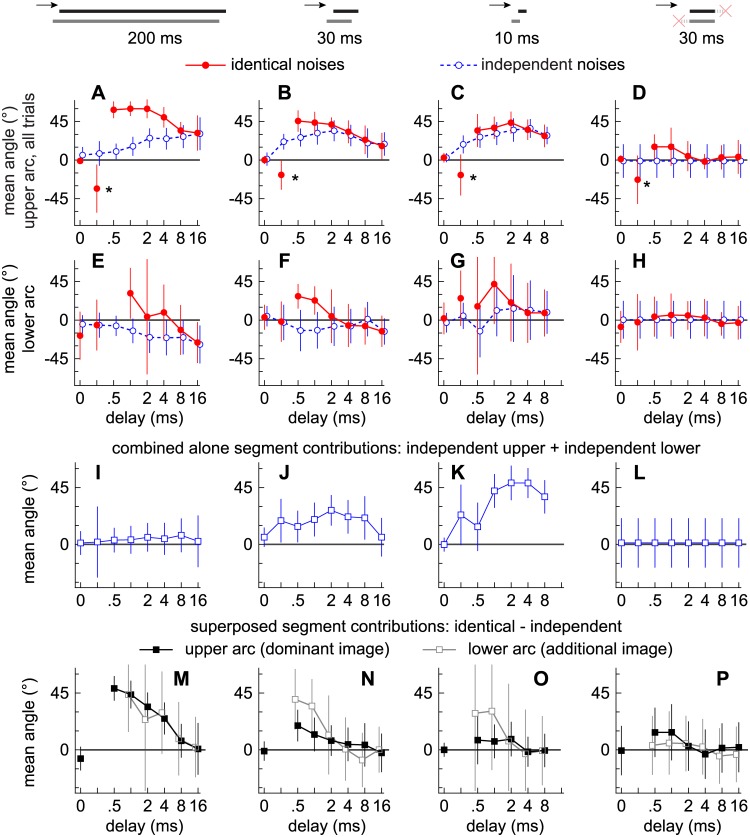
Contributions to localization dominance from the alone and superposed segments. Markers indicate the mean angles indicated by subjects on the upper **(A-D)** and lower **(E-H)** arcs at a given delay (abscissa) when the noise-pairs were 200 ms **(A** and **E)**, 30 ms **(B** and **F)**, 10 ms **(C** and **G)**, or 30 ms with synchronized onsets and offsets **(D** and **H)**. Error bars indicate variation across subjects (±1 s.d.). Solid lines and filled circles show the results for the identical noise-pairs. Dashed lines and open circles show the results for the independent noise-pairs. **(I-L)** Markers indicate the sums of the angles reported on the upper and lower arcs and thus the contributions to localization dominance from the alone segments. **(M-P)** Markers indicate the differences in the angles reported for the independent and identical noise-pairs on the upper (filled squares) and lower (open squares) arcs and thus the contributions to localization dominance from the superposed segment.

Fig 4A–4D and 4E–4H plot, respectively, the average locations indicated (μ) on the upper and lower arcs against delay. Each data point represents the average of the 10 subjects in each experiment (vertical lines show ±1 s.d.). When fusion is strong, an average near 0° indicates the presence a single mode near 0° (e.g., filled circles in Fig 2A, 2C and 2M). When two sources are reported, an average near 0° indicates the presence of two equal-sized modes straddling 0° (e.g., open circles in Fig 2A–2D and 2M). Positive μ values therefore indicate a higher incidence of responses on the leading side of an arc (e.g., circles in Fig 2I and 2K). Negative values indicate a higher incidence of responses on the lagging side (e.g., circles in Fig 2J and 2L). Angles corresponding to the distribution peaks (modes; Fig 2) are not reported because they lack precision when the auditory images were poorly circumscribed.

Results obtained with the independent noises (open circles) are first considered, from which contributions to localization dominance from the lead- and lag-alone segments are inferred. Contributions to localization dominance from the superposed segments are then inferred by comparing results for the independent (open circles) and identical (filled circles) noises.

#### Contributions to localization dominance from the alone segments

Contributions to localization dominance from the lead- and lag-alone segments may be inferred from the psychometric functions obtained with independent noises (open circles in Fig 4), since a superposed segment consisting of independent sounds is devoid of cues that could bias spatial perception toward either source. As shown below, evidence of localization dominance was therefore absent in Experiment 4 when the alone segments were excised from the independent noises.

**Fig 5:**
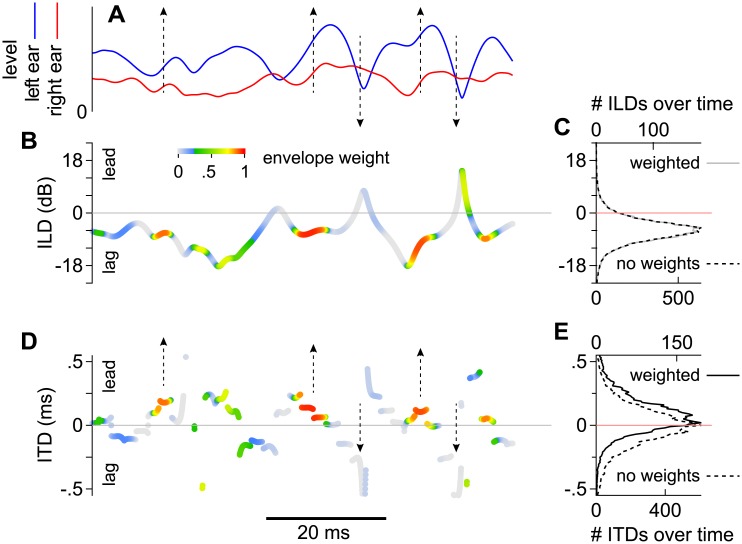
Demonstration of how ITD may be shifted toward the leading source (positively). **(A)** Amplitude envelopes of identical, 500 Hz, signals in the left and right ears when the lead-lag delay was 2.5 ms. **(B)** Measurements of ILD at the times when the signals were sampled, colored according to weights that were attributed to them by the envelopes of the left and right signals. **(C)** Distributions of weighted (solid line) and non-weighted (dashed line) ILDs measured when the leading and lagging noises pairs were superposed for 500 ms. **(D)** Measurements of ITD at the times when the signals were sampled, colored as in (B). **(E)** Distributions of weighted (solid line) and non-weighted (dashed line) ITDs measured when the leading and lagging noises pairs were superposed for 500 ms.

In Experiment 1, where the independent noises were 200 ms (open circles, Fig 4A), the average locations of the reports on the upper and lower arcs change in opposite directions with delay, with the dominant image being localized increasingly closer to the lead, (positively) and the additional image closer to the lag (negatively). Thus, as the alone segments lengthen, subjects indicate the two images at increasingly eccentric average loci. Importantly, the average location indicated for nonzero delays on the upper arc (μ_upper_; Fig 5A) is, on average, 4.1° farther from the midline (0°) than the average location indicated on the lower arc (μ_lower_; Fig 4E). In other words, both the dominant and additional images are biased toward the leading source. This is shown in Fig 4I, which plots against delay the sums of μ_upper_ and μ_lower_, thus: μ_upper arc_ + μ_lower arc_ = 4.1° (± 1.8°). As shown, the points are slightly shifted toward the lead (upward), suggesting weak localization dominance due to the presence of the alone segments.

The alone segments contributed considerably more to localization dominance when the (independent) noises were shortened to 30 or 10 ms in Experiments 2 and 3. Comparing the open circles in Fig 4B and 4F, it is clear that the average loci indicated on the upper arc at nonzero delays are, on average, farther from the midline than observed with the 200 ms noises, whereas those for the lower arc are closer to the midline. As a result, the dominant and additional images are shifted more toward the lead than was observed with the 200 ms noises. The sums of the distances from the midline, μ_upper_ + μ_lower_, are plotted against delay in Fig 4J and 4K. Thus, the contributions of the alone segments are greater when the noises are shorter. As expected, there were no biases toward either source when the alone segments were excised in Experiment 4 (Fig 4L).

#### Contributions to localization dominance from the superposed segment

Contributions to localization dominance from the superposed segments of the various stimuli (Experiments 1–4) were deduced for the dominant (Fig 4A–4D) and additional (Fig 4E–4H) images by comparing the psychometric functions obtained with identical (filled circles) and independent (open circles) noises. Thus, contributions to dominant image = μ_identical upper_—μ_independent upper_, and contributions to additional image = μ_identical_lower_—μ_independent_lower_.

When the noises were identical and the delay was 0 ms, subjects typically reported a single image near the center of the upper arc (μ_identical upper_ = 0°), a finding consistent with summing localization (Fig 4A–4D; see also Fig 2A, 2C and 2M). Note that at a delay of 0.25 ms (asterisks in Fig 4A–4D), which was the ITD with which the leading and lagging noises were spatialized (± 0.25 ms), subjects consistently reported an image on the side of the lagging source (μ_identical upper_ < 0). Under these latter conditions, analyses of binaural cues across a wide range of frequency bands showed that ILD consistently favored the lagging side (i.e., when delay = ±ITD), whereas ILD fluctuated with frequency and favored neither source at the longer delays [34, 35].

Functions obtained for the 200 ms identical noises (filled circles) are well above those for the independent noises (open circles) for delays between 0.5 and 8 ms (Fig 4A), demonstrating that localization dominance was considerably stronger for the identical noises. This is also apparent in Fig 4M (filled squares), which plots against delay the differences between the psychometric functions obtained with the independent and identical noises. The lead-ward bias diminishes with longer delays and the results with the independent and identical noises are indistinguishable at 16 ms.

For the shorter noise-pairs used in Experiments 2–4, differences between the identical and independent psychometric functions are smaller (Fig 4B–4D; filled squares in Fig 4N–4P). Thus, the superposed segment is seen to have contributed less to localization dominance when its length was shorter.

When additional images for the identical noises were reported on the lower arc, they too were shifted toward the lead in comparison to reports on the lower arc obtained with independent noises (Fig 4E–4H; open squares in Fig 4M–4P). Because fusion was often strong at the shorter delays (0.5–4 ms), additional images were infrequently reported across the subjects (200 ms: N = 199/2000 trials; 30 ms: N = 303/2000 trials; 10 ms: 346/2400 trials). Nevertheless, when the noises were 200 ms, the dominant and additional images were shifted by similar amounts (Fig 4M, open and filled squares). The additional image was shifted by an even greater amount, for some subjects, when the noise pairs were shorter (Fig 4N and 4O).

Taken together, the lengths of the alone segments, the lengths of the superposed segment, and stimulus similarity were each found to influence fusion and localization dominance.

## Neural Model

Below, a neural model consistent with spatial perception under precedence conditions is examined. We first examine the extent to which the envelopes of filtered stimuli may explain localization dominance [27, 36]. This is followed by an analysis of binaural cues generated during the various stimulus segments and a description of how fluctuations in spatial cues may explain fusion if represented by sub-maximal activities across spatially selective neurons [10–13].

### Envelopes and spatial cue distributions

Studies of the barn owl have suggested that low-frequency (40–150 Hz) envelopes imposed on lead-lag stimuli, lacking lead or lag-alone segments, can invoke neural responses and behaviors consistent with localization dominance [27, 36]. A key aspect of these findings was that spatially selective neurons were found to respond more frequently when the experimenter-imposed envelopes of the leading and lagging stimuli (*Ē*) were both rising (*dĒ/dt* > 0) than when they were declining (*dĒ/dt* < 0). Similar findings have since been observed for binaural beat stimuli [37–39].

Here, a similar model is proposed for noises bursts that contain only intrinsic amplitude modulations and no experimenter-imposed modulations. Even for such noises, the outputs of low-frequency (<1.5 kHz) cochlear filters are slowly (< ~150 Hz) and deeply amplitude-modulated. Shown in Fig 5A, for instance, are the envelopes of representative broadband noises passed through filters simulating the left (l) and right (r) cochlea at 500 Hz [40]. The envelopes of the signals, obtained using the Hilbert transformation, differ in level and shape because a 2.5 ms delay was introduced between the leading and lagging stimuli that were spatialized with ITDs of 250 and -250 μs, respectively, but were otherwise identical. As noted by Dizon and Colburn [26], this stimulus configuration, at 500 Hz, is interesting because significant ILDs are produced over time (Fig 5B) that favor the lagging source’s location on average. This can be seen in Fig 5C, which shows the distribution of ILDs accumulated over the stimulus’ duration (mean ILD ≈ -6.5 dB). Yet subjects in the study of Dizon and Colburn [26] did not always lateralize an intracranial image on the side of the lagging stimulus. Instead, they frequently lateralized the image on the opposite side, as if another cue were biasing their perception toward the leading source. Similar biases were shown for other frequency bands (< ~2 kHz) and other delays within the precedence range, regardless of ILD. Thus, modulations intrinsic to cochlear filters may be responsible for some aspects of the precedence effect when the bandwidths of superposed stimuli are wide enough.

An analysis of ongoing ITDs (Fig 5D) and ILDs (Fig 5B), obtained from Hilbert transformed signals in the ears, provides a possible explanation if spatially selective neurons in humans, as in the owl, are presumed to discharge when the envelopes of the signals in the ears are rising. ITDs were obtained by subtracting the phases of the signals after dividing them by frequency. ILDs were obtained by subtracting the envelopes of the transformed signals. When the envelopes of the signals are rising (upward arrows in Fig 5A), representative ITDs in Fig 5D are mostly positive (toward the leading source) and neurons responsive to these ITDs may be more likely to fire. In contrast, when the envelopes are declining (downward arrows in Fig 5A), the representative ITDs are mostly negative (toward the lagging source), and neurons responsive to these ITDs may be less likely to fire.

How ITDs are distributed over time and possibly ‘weighted’ by amplitude modulations further demonstrates why ITD may favor the leading source under precedence conditions. The dashed line in Fig 5E shows the average distribution of ITD over a time period of 15 seconds (30 different stimuli that were each 500 ms). As described by Dizon and Colburn [26], the distribution’s central tendency is close to zero. However, when weights representing envelope derivatives are applied (see below), as shown by the solid line, the distribution’s central tendency and skew shifts toward the leading source (positively). In contrast to ITD, distributions of ILD remain roughly the same whether they are weighted or not (overlapping solid and dashed lines in Fig 5C). Note that ILD would be biased in a way resembling ITD if the stimuli were spatialized using ILD instead of ITD, and that distributions for both cues may often be biased if the stimuli were spatialized using both cues (not shown).

**Fig 6:**
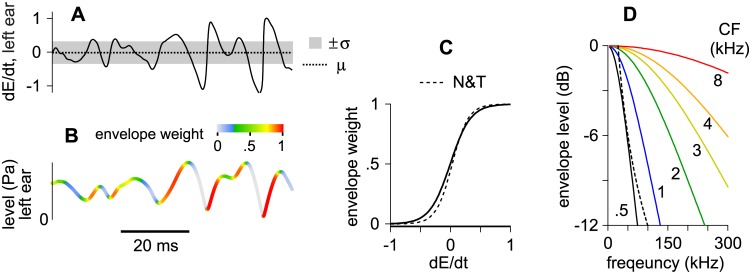
Demonstration of how envelope weights were obtained from band-filtered noise-pairs. **(A)** Envelope derivatives measured from the envelope of the signal in the left ear. A horizontal dashed line shows the mean derivative (μ) whereas gray shading shows ±1 standard deviation (±σ). **(B)** Envelope of the signal in the left ear from which the derivatives were measured. Shading shows the weights that were attributed to the signal using Eq 1. **(C)** Weights calculated using Eq 1 (solid line) for a continuous range of dE/dt values (abscissa). The dashed line shows normalized weights derived for the barn owl from the responses of space-map neurons to the derivatives of deeply amplitude-modulated noise bursts. **(D)** Spectra obtained from the envelopes of band-filtered noises (0.5–8 kHz; solid lines). A single dashed line, resembling the 500 Hz band, shows the average spectrum of the low-pass envelopes that were examined by Nelson and Takahashi [27, 36]. Note that weights used in the owl study (dashed line in C) are similar to those at 500 Hz (solid line in C) because their envelope spectra (D) are similar.

To obtain envelope weights across sampled points (44.1 kHz), the leading and lagging signals were first summed individually for the left (l) and right (r) ears (earphones). The resulting signals were then passed through a bank of 64 gammatone filters with center frequencies (f) between 0.2 and 1.5 kHz [40]. Envelopes (E) for each ear and frequency band were next obtained and derivatives (dE/dt) were measured across adjacent points of the sampled envelopes. Weights (Wt), which are presumed to represent the probability of a neuron firing (see below), were then calculated as:

$$Wt=1/1+e^{-(dE/dt-\mu )/\sigma }$$ (1)

where μ was the average derivative observed while the stimuli were superposed within a given frequency band (horizontal dashed line in Fig 6A; usually very close to zero) and σ was the standard deviation (shaded region in Fig 6A). Weights calculated for the 500 Hz band, in the left ear, are shown for a continuous range of dE/dt values by the sigmoidal function in Fig 6C (solid line). Analogous weights were calculated for the right ear using the same equation and values of μ and σ that were specific to this ear’s envelope (not shown). Weights derived for the left and right ears were then multiplied and are shown by hot colors in Figs 5B, 5D and 6B. For comparison, the dashed line in Fig 6C shows the analogous sigmoidal function (Fig 3B in [36]), derived for the barn owl from the responses of space-map neurons to the derivatives of deeply amplitude-modulated noise bursts [27]. To our knowledge, this implementation is novel in that no experimenter-imposed modulations are necessary and no modulations were imposed on the stimuli.

Selectivity for the rising edges of envelopes may be limited by several factors, including a decline in the responses of inferior colliculus (IC) neurons to high-frequency amplitude modulations. The envelope of a noise filtered at 2 kHz, for instance, can contain significant energy above ~200 Hz (Fig 6D) but these components may be removed by a typical ITD modulation transfer function [41], causing the envelope to effectively flatten. The envelopes of identical lead-lag stimuli are also expected to become effectively dissimilar (independent) when they occur with a period that is shorter than the stimulus’ lead-lag delay [27, 36]. Significant envelope energy above 100 Hz (Fig 6D), for instance, may cause dissimilar envelope features to overlap in time, instead of similar features, when the lead-lag delay is longer than ~10 ms (~100^−1^ Hz). In terms of the stimuli after they sum in the ears (Fig 5A), this means that the envelopes of filtered signals may be declining (downward arrows in Fig 5A) as often as they are rising (upward arrows in Fig 5A) when spatial cues favor one source or the other (e.g., Fig 5D). Consistent with this interpretation, precedence phenomena weaken at short delays when high frequency noise-bands are tested [26].

**Fig 7:**
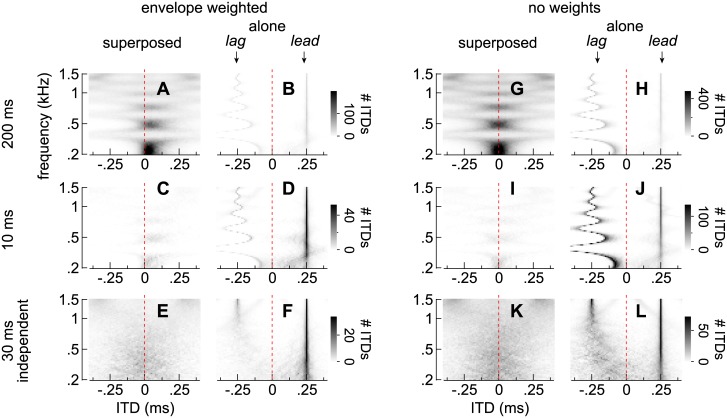
Frequency-specific distributions of ITD when the lead-lag delay was 4 ms. **(A-F)** Distributions of envelope-weighted ITDs attributed to the superposed and alone segments when identical noise-pairs were 200 or 10 ms (**A**-**B** and **C**-**D**, respectively) or when the noise-pairs were 30 ms and independent (**E**-**F**). Each distribution shows the average of 100 different noise-pairs and reflects cues observed only during the superposed segment **(A, C,** and **E)** or only during the alone segments **(B, D,** and **F)**. Note that the same gray-scale is applied to each stimulus and pair of plots. **(G-L)** Distributions of *unweighted* ITDs generated by the same noise-pairs and segments as in **(A-F)** (see labels above and to the left of the plots).

Frequency-specific distributions of ITDs, attributed to the superposed and alone segments of noise-pairs as described in S1 Fig, are shown in Fig 7 *with* (A-F) and *without* envelope weights (G-L). Gray-scale bars show the frequency of occurrence of ITD values in a given frequency band, averaged over 100 different noises pairs.

Comparing plots on the left (A-F) and right-hand (G-L) sides of Fig 7 shows the effects of envelope *weighting* when the lead-lag delay is 4 ms. As in Fig 5E, ITDs arising during the superposed segments of the identical noise-pairs shift and skew toward the leading source (positively; e.g., compare Fig 7A and 7G). This effect is absent, however, when independent noise pairs are considered (e.g., compare Fig 7E and 7K).

During the alone segments, envelope weights similarly emphasize the ITDs of the leading source over those of the lagging source. This is most visible for the shorter noise-pairs. Thus, distributions of ITDs attributed to the lead-alone segment (distribution peaks ≈ 0.25 ms) in Fig 7D are higher (darker) than those that oscillate with frequency and are attributed to the lag-alone segment (distribution peaks < 0 ms). Without weights, the distributions have similar heights, as one can see in Fig 7J. Under our model, this difference occurs because amplitude always rises at the onset of the lead-alone segment but generally declines during the lag-alone segment (S1 Fig). This effect also occurs for independent noise-pairs (e.g., compare Fig 7F and 7L).

Importantly, cues accumulated during the alone segments are unrelated to a given noise pair’s length and are determined instead by the stimulus’ lead-lag delay. Thus, differences in cues observed as the noise-pairs are lengthened, as well as differences in perception (Figs 2–4), may not be attributable to the alone segments, but instead, to differences in cues accumulated across the superposed segment.

Under our model, weights were accumulated over the entire lengths of the various stimulus segments because stimulus duration was found to have a considerable influence on subject’s responses (e.g., Fig 3). Our application of envelope weights (Figs 5 and 6) also means that cues were effectively integrated over much shorter, frequency-specific, time-scales (see Discussion section: Effects of time, envelopes, and spatial cue distributions).

#### Edge hypothesis

**Fig 8:**
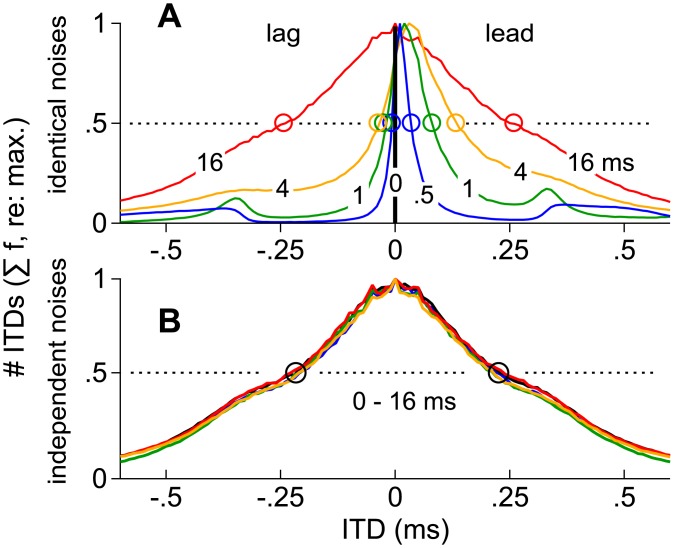
Distributions of ITD averaged across frequency and the superposed segments of noise-pairs. (**A**) Distributions of ITD, averaged across frequency (0.5–1.5 kHz) and across the superposed segments 100 different, 200-ms, noise-pairs, when the lead-lag delay was 0, 0.5, 1, 4, or 16 ms. Because each distribution is scaled to a maximum of 1.0, open circles indicate the ITDs that occurred at the 50% point along the edges of the distributions. **(B)** Distributions of ITD as described in (A) but for independent noise-pairs.

When there is no delay between a pair of identical noises, the stimuli are diotic and spatial cues equal the averages of the left and right sources. At delays less than approximately 1-ms, subjects experience summing localization. As the delay increases, distributions of envelope-weighted ITDs during the superposed segment not only shift and skew toward the leading source (positively), but also broaden in a delay-dependent manner. These differences are shown in Fig 8A for the superposed segments of 200-ms noise-pairs lacking alone segments. To simplify presentation, frequency-specific distributions, as shown for a 4-ms delay in Fig 7A, 7C and 7E, were averaged across frequency and 100 different noise-pairs.

The distributions broaden with delay (Fig 8A) and each stimulus generates only a single mode. Why then do subjects often report one image (Fig 3A) when identical noise-pairs are tested at short delays (blue, green, and orange lines in Fig 8A) but two images (Fig 3A) at long delays (red line in Fig 8A) or when independent noise-pairs are tested (Fig 8B)? One possibility is that the subjects’ reports may reflect the neural representations of the edges of the distributions, not their central tendencies. Specifically, ITDs corresponding to the edges of the distributions, defined operationally as the 50% point on the ordinate of (Fig 8), for instance, are identical when there is no delay (dark vertical line at 0-ms), a condition that could lead to summing localization were subjects reporting ITDs corresponding to the distribution’s edges. This would also be the case when there is but a single source. The edges (open circles in Fig 8A) then diverge as the delay increases until points corresponding to 50% are separated by ~0.4 ms of ITD (16 ms delay; red line) or when the noise-pairs are independent (Fig 3B), at which point subjects’ responses evince “fission” i.e., a tendency to report two sources (Fig 3A and 3I). The edges are also seen to shift toward the ITD of the leading source (positively) at intermediate delays, a finding consistent with localization dominance. Lastly, note that distributions obtained at the longest delay (16 ms) are nearly identical to those generated by independent noise-pairs, regardless of delay (Fig 8B).

We next consider how the edges of ITD distributions may be represented across subpopulations of ITD selective neurons and the degree to which these representations, when combined with envelope weighting, may explain spatial perception and reproduce our psychophysical findings (Figs 3 and 4).

#### Neural Representations of Cue-Distributions

How might the edges of ITD distributions be represented in the auditory system? One possibility is that spatial perception could correspond to intermediate or sub-maximal activities evoked across spatially selective neurons, as suggested in a number of previous studies [10–13]. Although these studies only addressed single sources, when spatial cues would be narrowly distributed, they pointed out that the ipsilateral slopes of individual tuning curves often correspond to frontal locations, where spatial acuity in humans is greatest, whereas their maxima correspond to contralateral locations [4, 5]. In addition, ipsilateral slopes may be steeper than their contralateral counterparts [3] allowing them to convey greater information than their peaks [42, 43]. Note that this sub-maximal hypothesis [10–13] differs markedly from the hypothesis that spatial locations may be represented by differences in activities evoked across neurons throughout the left and right hemispheres [1, 4, 5].

The model only consider frequencies below 1.5 kHz since effects of envelope weights are expected to diminish at higher frequencies and because earlier studies have shown that precedence phenomena are strongest at low frequencies [26, 35, 44, 45]. Frequency-specific neurons with best interaural phase differences (IPD) beyond the “π-limit” [4, 5] are also considered, so that the implications of this limit may be examined under the edge model.

**Fig 9:**
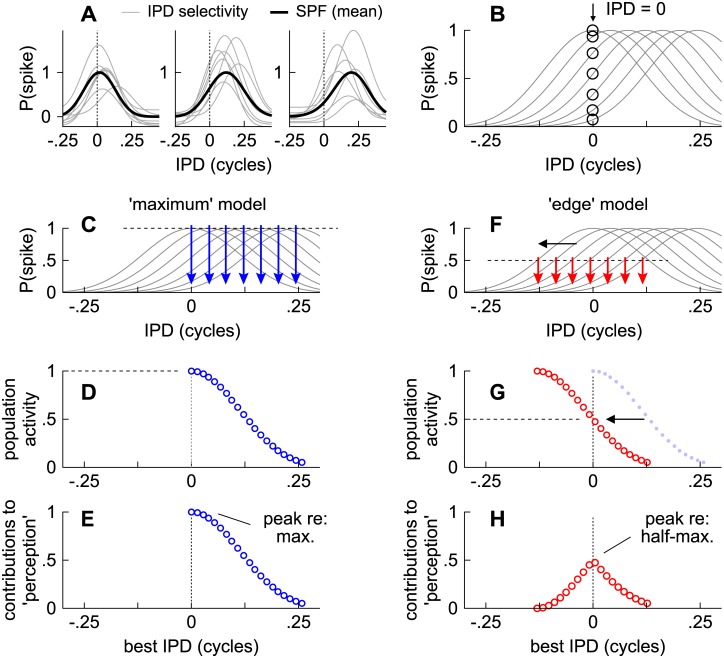
Description of the edge model. (**A**) Thick black lines show three different spike probability functions (SPF), each reflecting the average preference of a subpopulation of integrated neurons (thin gray lines). (**B**) Hypothetical array of SPFs in the left IC (thin lines). Open circles indicate the probabilities of spiking across the SPFs when the stimulus’ IPD is 0 cycles. (**C**) Array of SPFs in which downward arrows indicate the IPD at each SPF’s maximum. (**D**) Probability of spiking across as array of SPFs, where the abscissa indicates the IPD at each SPF’s maximum. (**E**) Contributions to perception from an activity maximum centered at an IPD of 0 cycles. (**F**) Array of SPFs in which downward arrows indicate the IPD at each SPF’s half-maximum. (**G**) Probability of spiking across as array of SPFs, where the abscissa indicates the IPD at each SPF’s half-maximum. (**H**) Contributions to perception from an activity half-maximum centered at an IPD of 0 cycles.

To simulate activities across each IC, stimulus-specific distributions of IPD were convolved with the “spike probability function” (SPF), which represents the probabilities that an IPD (*Φ*) at a given moment in time would evoke a single spike in a subpopulation of IPD-selective neurons. Fig 9A shows three such subpopulations. Each thin gray line is the IPD “tuning curve” of a single neuron in the IC. Heavy black lines represent their averages, where the maximum probability of a spike corresponds, for instance, to an IPD of ~0 cycles (left), ~0.15 cycles (center), or ~0.2 cycles (right). Subpopulations of neurons are considered because the firing rates of individual neurons are too low to represent IPD on the time-scales of amplitude modulations intrinsic to cochlear filters (e.g., Fig 6B). Note that it is not necessary to assume any topography, in the representation of IPD, such as on an auditory space map. The shape of the SPF is assumed to resemble a typical IPD tuning curve, as described by Harper and McAlpine [43]:

$$\text{P}(\text{spike})\;=\;{\left[\frac{1}{2}+\frac{1}{2}cos(\phi )\right]}^{4}$$ (2)

To describe the ‘edge’ model and distinguish it from the more conventional ‘maximum’ model (e.g., [2]), an array of SPFs is first considered (lines in Fig 9B). The peaks of the SPFs correspond to positive IPDs within the π-limit (<0.25 cycles) because they represent the activities of neurons in the left IC. Open circles indicate the probabilities of spiking across the SPFs when the stimulus’ IPD is 0 cycles.

Under the 'maximum' model, spatial perception is assumed to correspond to activity maxima across the IC. Thus, Fig 9D reflects the output of an array of SPFs in which the ordinate indicates the probability of a spike (see Fig 9B) and the abscissa indicates the IPD that corresponds to each SPF’s maximum (downward arrows in Fig 9C). Accordingly, perception would be based on the single activity maximum centered at an IPD of 0 cycles (Fig 9E).

In contrast to the ‘maximum’ model, spatial perception under the ‘edge’ model is determined by neurons firing at sub-maximal values. Thus, Fig 9G reflects the output of the same array of SPFs as in Fig 9D except that the abscissa indicates the IPD that corresponds to each SPF’s half-maximal spike probability (downward arrows in Fig 9F), as opposed to their maxima (Fig 9C). Maximal spike probabilities may not contribute as much to spatial perception under the edge model and are therefore reflected downward in Fig 9H, resulting in a single half-maximal ‘peak’ at 0 cycles (at 0.5 along the ordinate).

**Fig 10:**
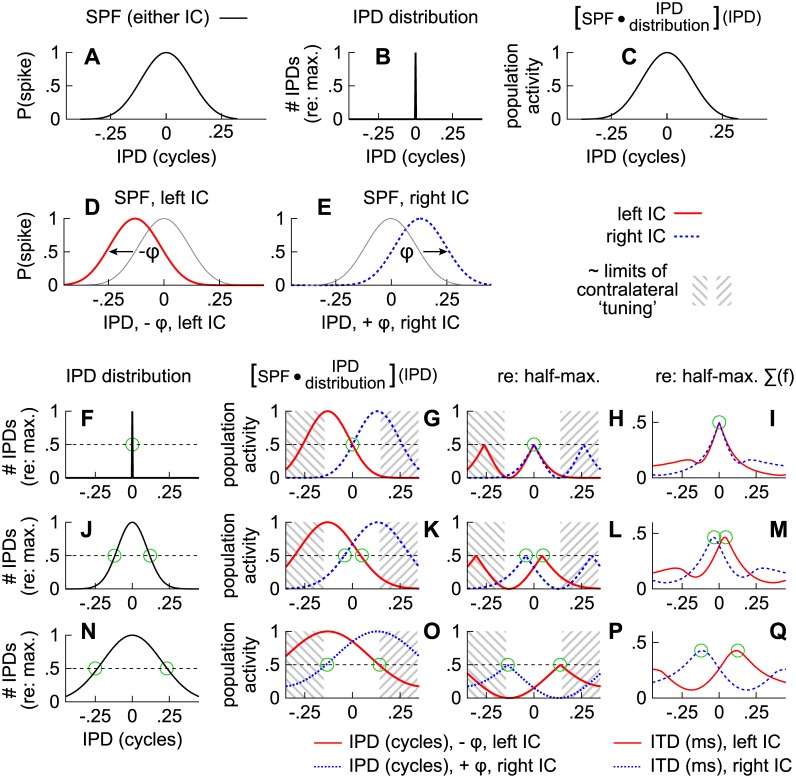
Computationally efficient implementation of the edge model. (**A**) Spike probability function (SPF) corresponding to the maximum model. (**B**) Narrow distribution of IPDs. (**C**) Results of convolving the SPF in (A) with the distribution of IPDs in (B). (**D**) Spike probability function for the left IC under the edge model. (**E**) Spike probability function for the right IC under the edge model. (**F**) Narrow distribution of IPD for which an open circle indicates the IPD that arose half-maximally. (**G**) Modeled population activities when the distribution of IPD is narrow (F). Cross-hatching indicates activities of neurons with best IPDs beyond the ‘pi-limit’ (< -0.13 or > 0.13 cycles) which may rarely occur (e.g., [3, 4]) and therefore contribute little to spatial perception. (**H**) Relative contributions of population activities to spatial perception under the edge model. (**I**) Modeled activities obtained after converting IPD to ITD and averaging across frequency to simplify presentation (0.2–1.5 kHz). **(J-Q**) Distributions of IPD and modeled activities as described in (F-I) but when the distributions of IPD (J and N) were broader.

To infer activities across both ICs in a computationally efficient manner, SPFs and stimulus-specific distributions of IPD were convolved (Fig 10A–10C). Fig 10A and 10B show, respectively, an SPF and a narrow distribution IPDs (mean, standard deviation = 0). Fig 10C shows the result of the convolution. Note that the traditional ‘maximum’ model is assumed in this example and that similar results are expected for the left and right ICs.

To implement the edge model, SPFs representing the left and right ICs were phase-shifted (±φ) by an amount equal to the distances from their maxima to their ipsilateral (inner) half-maxima (φ = ±0.13 cycles) thus:

$${\text{P(spike)}}_{\text{left IC}}\;=\;{\left[\frac{1}{2}+\frac{1}{2}cos(\phi +\varphi )\right]}^{4}$$ (3)

$${\text{P(spike)}}_{\text{right IC}}\;=\;{\left[\frac{1}{2}+\frac{1}{2}cos(\phi -\varphi )\right]}^{4}$$ (4)

These phase-shifts indicate neither a shift of activity within each IC nor differences in spatial “tuning” per se. Instead, they represent the notion that spatial locations may be represented by subpopulations of neurons (SPFs) whose firing probabilities are half of other neurons throughout the left and right ICs (Fig 9F–9H). This is conceptually similar to labeling a single neuron’s “tuning curve” according to the cues at which its firing *rates* are half-maximal (Fig 9F), as opposed maximal (Fig 9C), except that the SPF reflects the momentary probability of spiking across many neurons (Fig 9A).

When distributions of IPDs are narrow, as in Fig 10F, modeled population activities (Fig 10G) mirror the SPFs (Fig 10D and 10E) and half-maximal activities in each IC correspond to the same IPD (open circle in Fig 10G). Note that the two distributions in Fig 10G would have both been centered on 0 cycles, as shown in Fig 10C, were the SPFs referenced to their maxima (Eq 3) rather than their ipsilateral half-maxima (Eqs 4 and 5).

The relative contributions of ITD to spatial perception are assumed to be greatest when population-wide activities are half-maximal. Conversely, the contributions are assumed to be lowest when activities are either lower (activity < 0.5) or higher (activity > 0.5) than the half-maximum. To better visualize contributions to spatial perception, activities greater than the half-maximum (>0.5) are therefore reflected downward in Fig 10H. Thus, half-maximal “peaks” near 0.5 in Fig 10H indicate the IPDs that are expected to contribute most to spatial perception. Note that significant half-maximal side-peaks are evident near ±0.25 cycles but may be diminished under at least two conditions. The first is if listeners were to integrate across frequency, as shown in Fig 10I, where IPD was converted to ITD and frequency-specific activities, analogous to those in Fig 10H, were averaged (0.2–1.5 kHz). Half-maximal side peaks could also be diminished if neurons “tuned” (maximally) to ipsilateral IPDs were excluded from the convolutions along with neurons tuned (maximally) to IPDs beyond the π-limit, both of which are equivalent to excluding half-maximal activities less than approximately -0.13 or greater than 0.13 cycles (cross-hatching in Fig 10).

Besides offering a higher spatial resolution when spatial cues are narrowly distributed over time, owing to the steep slopes of the modeled SPFs, distinct images are predicted in the left and right ICs when IPDs (or ITDs) are broadly distributed. This is shown in Fig 10 where IPD distributions having standard deviations of 0.1 cycles (Fig 10J–10M) or 0.2 cycles (Fig 10N–10Q) were considered. After convolving these broader distributions (Fig 10J and 10N) with the SPFs (Fig 10B and 10C), activities in both ICs are predicted to broaden (Fig 10K and 10O), causing half-maximal values to be achieved at distinct IPDs in each IC (Fig 10L and 10P). Fig 10M and 10Q show again that ITD half-maximal side-peaks can be significantly diminished if listeners are able to integrate across frequency (0.2–1.5 kHz) or if half-maximal activities are constrained to between approximately -0.13 cycles and 0.13 cycles (cross-hatching in Fig 10), due to a paucity neurons tuned (maximally) to ipsilateral IPDs or IPDs beyond the π-limit.

**Fig 11:**
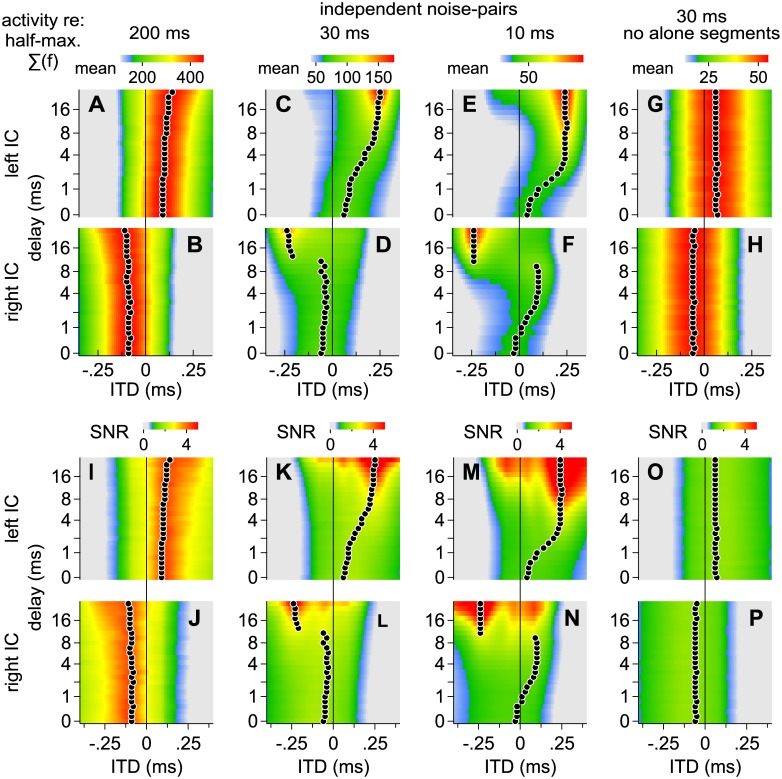
Modeled contributions to spatial perception for *independent* noise-pairs of various lengths and lead-lag delays. Warm colors along each row represent ITDs expected to contribute most heavily to spatial perception, on average, across listening trials in a given IC. For each row and lead-lag delay, a circle indicates the ITD at which activities were half-maximal. (**A** and **B**) Contributions to perception in the left and right ICs, respectively, when the noise-pairs were 200 ms. (**C-H**) Contributions as indicated for the left and right ICs in A and B but when the noise-pairs were 30 ms (C and D), 10 ms (E and F) or 30 ms and lacking alone segments (G and H). (**I** and **P**) Signal-to-noise ratios (SNR), computed as the ratio of half-maximal activity over one standard deviation, when the noise-pairs were 200 ms (I and J), 30 ms (K and L), 10 ms (M and N) or 30 ms and lacking alone segments (O and P). Warm colors indicate a higher SNR.

**Fig 12:**
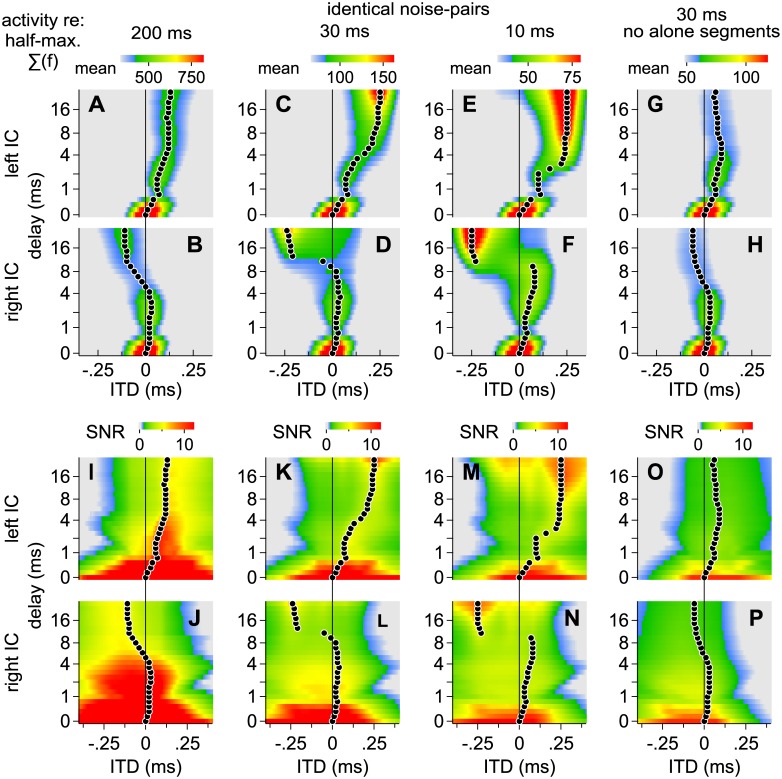
Modeled contributions to spatial perception for *identical* noise-pairs. See Fig 11 and the text for descriptions of the various plots.

Distributions of envelope-weighted ITDs can broaden significantly when independent noise-pairs are considered or when long delays are introduced between identical lead-lag noise-pairs (Figs 7 and 8). Figs 11 and 12, reflecting independent and identical noise-pairs, respectively, therefore show modeled results as a function of lead-lag delay (ordinate axis), when distributions of ITD over time were: (1) convolved with SPFs representing ITD as opposed to IPD, (2) reflected so that greater values (warmer colors) indicate half-maximal activities, and (3) integrated across frequency to simplify presentation, as illustrated in Fig 10. In addition, the modeled results were obtained for 100 different noise-pairs and averaged. Warm colors along each row represent ITDs expected to contribute most heavily to spatial perception, on average, across listening trials in a given IC (A, C, E, G = left IC; B, D, F, H = right IC). For each row and lead-lag delay, a circle indicates the ITD at which activities were half-maximal.

Signal-to-noise ratios (SNR), computed as the ratio of half-maximal activity (warm colors in Figs 11A–11H and 12A–12H) over one standard deviation (100 different noise-pairs), are shown in Figs 11I–11P and 12I–12P. A high SNR (warm color) indicates a pattern of activity that, on any single trial, is likely to resemble the average pattern (Figs 11A–11H and 12A–12H). In contrast, a low SNR indicates a pattern of activity that, on any single trial, may only rarely resemble the average pattern. In other words, the numerator (signal) represents the pattern of activity observed across trials whereas the denominator (noise) represents variation across trials. High SNRs are generally observed when ITDs are consistent over time, such as when identical noise-pairs with short lead-lag delays are considered (Fig 12I–12P) or when the lead or lag-alone segments are long (Figs 11I–11P and 12I–12P). SNRs also increase with stimulus duration. Thus, at short and intermediate delays, SNRs were higher for the longer, 200 ms, stimuli than for the shorter, 30 or 10 ms, stimuli.

## Qualitative and Empirical Predictions

The precedence effect is characterized by empirical measures, such as fusion and localization dominance (Figs 2–4). The results of our model, summarized in Figs 11 and 12, should therefore predict these empirical observations, provided that the model’s output is interpreted in terms of the psychoacoustical task and the manner in which the data were analyzed.

### Fusion

Fusion, which is the tendency of listeners to report a single auditory image, is strongest when stimulus pairs are identical and the lead-lag delay is short. Our model is consistent with this characterization in that similar half-maximal activity patterns are predicted in the left and right ICs, both corresponding to ITDs near 0 ms (Fig 12). In contrast, activity patterns in the left and right ICs are often distinct, corresponding to different ITDs, when the lead-lag delay is long (Figs 11 and 12) or when the stimulus pairs are independent (Fig 11).

**Fig 13:**
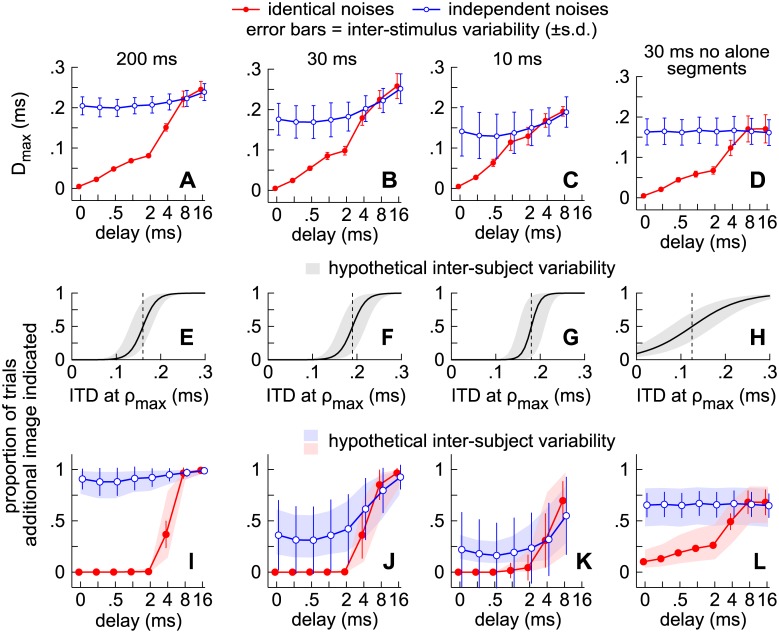
Empirical predictions of fusion. (**A-D**) Correlogram maxima (D_max_) describing the degree to which activities in the left and right ICs corresponded to distinct ITD. (**E-H**) Sigmoidal functions used to predict the proportions of trials (**I-L**) in which an additional image was reported.

To model our empirical results (Fig 3A–3D), half-maximal activities derived for the left and right ICs were averaged across frequency (see Figs 11 and 12) and cross-correlated. The degree to which activities in each IC correspond to distinct ITDs was then inferred from the time lag at the correlogram’s maximum (D_max_, Fig 13A–13D). Sigmoidal functions (Fig 13E–13H) were next used to predict the proportions of trials in which an additional image was reported (Fig 13I–13L), thus:

$$\text{proportion additional image}=\left[1/1+e^{(M-D_{\text{max}})/R}\right]$$ (5)

where values of M and R were chosen to provide reasonable fits to our psychophysical results (Fig 3; Exp. 1: M = 0.00016, R = 0.000015; Exp. 2: M = 0.00019, R = 0.000015; Exp. 3: M = 0.00018, R = 0.00001; Exp. 4: M = 0.000125, R = 0.000055). Different values of M and R were used for each experiment because different subjects were tested (with one exception). Criteria used by the subjects to judge when an additional image was heard may also have differed across the four experiments. In comparison to Experiment 2 (Fig 3B), for example, subjects in Experiment 4 (Fig 3D) were more likely to report an additional image at short delays. We presume this difference is because the alone segments were excised in Experiment 4 and subjects may therefore have used less stringent criteria in deciding when an additional image was heard.

In most major respects, the modeled proportions (Fig 13I–13L) resemble our behavioral results (Fig 3A–3D). When the noise-pairs were identical, for instance, both proportions rose for delays longer than ~2 ms as is evident by comparing the filled circles and solid lines in Fig 3A–3C with those in Fig 13I–13K. Both proportions also remained consistently high for the longer (200 ms) independent noise-pairs, regardless of delay (open circles and dashed lines in Figs 3A and 13K) but declined at shorter delays (<~4 ms) when shorter noise-pairs were tested or modeled (30 or 10 ms; Figs 3B, 3C, 13J and 13K).

Contributions to fusion from the alone and superposed segments may also be inferred. Contributions from the alone segments, for instance, may be deduced from the proportions obtained with independent noises (open circles in Fig 13I–13K). Similarly, contributions from the superposed segment may be deduced by comparing the proportions obtained for the identical (filled circles) and independent noises (open circles). For example, comparing Figs 13I and 3A in which the noise-pairs were 200 ms shows that in both cases the proportions are considerably higher for the independent noises (open circles and dashed lines) than for the identical noises (filled circles and solid lines), suggesting that corresponding features during the identical stimulus’ superposed segment (~200 ms) contributed substantially to fusion. In contrast, the proportions for the identical and independent noise-pairs are more similar when shorter noise-pairs were tested in both the modeled and empirical results (Figs 13J, 13K, 3B and 3C), suggesting that the superposed segment contributes less to fusion as it is shortened.

Inter-subject variability (vertical error bars in Fig 3) may also be accounted for if we consider inter-stimulus variability (vertical error bars in Fig 13) and assume that subjects may have differed in their propensities to report an additional image when half-maximal activities in the left and right ICs differed. To simulate this latter effect, the midpoint at which subjects were predicted to indicate an additional image in 50% of the trials (M in Eq 6) was varied in a uniform manner (between ± 0.05), resulting in the gray-shaded regions shown in Fig 13E–13H. Lightly colored shading in Fig 13I–13L show the results. When D_max_ values are consistently large (independent noises, open circles in Fig 13A) or small (identical noises and short delays, filled circles in Fig 13A–13D), the model predicts only a moderate degree of inter-subject variation, as was observed when similar noise-pairs were tested (Fig 3A–3D). When D_max_ values are intermediate, however, greater inter-subject variation is predicted, as shown by broader shading in Fig 13I–13L.

### Localization dominance

Localization dominance, which is the tendency of listeners to report a single image or pairs of images closer to the source of the leading sound, is strongest when identical stimuli are considered and the lead-lag delay is short. Our model is consistent with this characterization in that distributions of ITDs (Figs 7A–7F and 8A) and half-maximal activity patterns (Figs 11 and 12) are predicted to ‘shift’ toward the leading source (positively) under such conditions. In contrast, the shift is diminished at long delays (Fig 12) or when noise pairs are independent (Fig 11).

**Fig 14:**
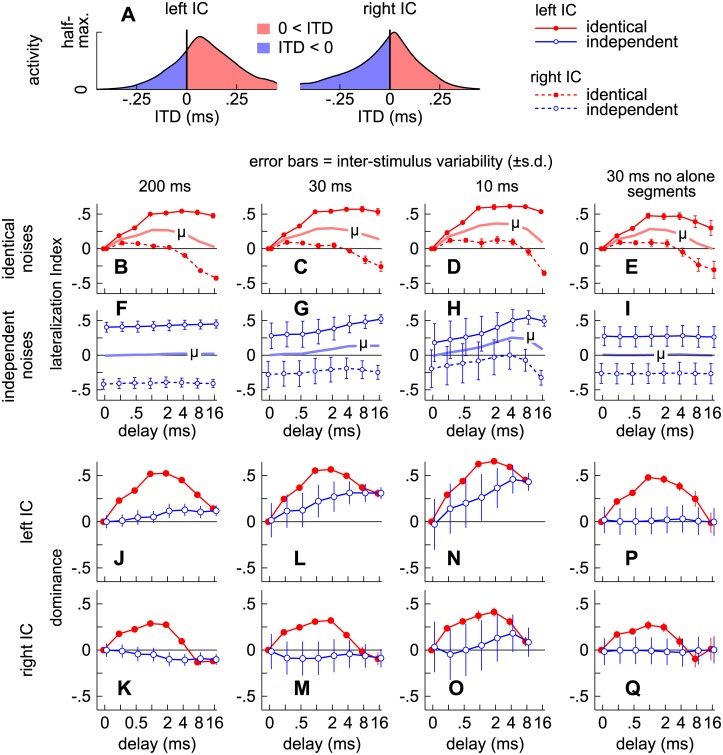
Empirical predictions of localization dominance. (**A**) Modeled half-maximal activities, averaged across frequency, in the left and right ICs when the lead-lad delay was 4 ms and the noise-pairs were identical. (**B-I**) Lateralization indices (*LI*) indicating the degree to which half-maximal activities in the left and right ICs correspond to leading or lagging (positive or negative) ITDs after averaging across frequency. (**J-Q**) Empirical predictions of localization dominance obtained as a product factors described by Eq 7.

To quantify these shifts, we computed lateralization indices (*LI*), which indicate the degree to which half-maximal activities in the left and right ICs correspond to leading or lagging (positive or negative) ITDs after averaging across frequency. Each index quantifies the proportion of half-maximal activities corresponding to positive ITDs (red areas in Fig 14A), thus:

$$LI=\left(\sum_{ITD=0}^{0.5}act-\sum_{ITD=-0.5}^{0}act\right)/\sum_{ITD=-0.5}^{0.5}act$$ (6)

where *act* represents neural activity relative to half-maximum (as plotted in Fig 10H, 10L and 10P). Indices for the left and right ICs (dashed and solid lines respectively) are plotted in Fig 14 for identical (B-E) and independent (F-I) noise pairs.

Because localization dominance can apply to conditions when one or two images are reported, our empirical results (Fig 4A–4H) must be simulated as the product of several additional factors:

$$\text{dominance }=LI_{fusion}•\text{ uncertainty }•\text{ symmetry}$$ (7)

The first term on the right side of the Eq 7, *LI*_*fusion*_, takes into account that the left and right ICs may represent distinct auditory locations when fusion is weak but a single location when fusion is strong. Values of *LI*_*fusion*_ are thus obtained by weighting *LI* (Eq 6) with the probability of an additional image having been reported (*P*, Fig 13I–13L):

$$LI_{fusion}=((LI•P+1)+(LI_{\mu }•(1-P)+1)/2$$ (8)

where *LI*_*μ*_ is the average of the left and right IC lateralization indices (lines without symbols in Fig 14B–14I). When *P* is very low (strong fusion), left and right *LI*_*fusion*_ values equal *LI*_*μ*_ and each other. When *P* is high, *LI*_*fusion*_ values differ for the left and right ICs and equal their respective *LI* values (circles in Fig 14B–14I).

The second term in Eq 7, *uncertainty*, considers that subjects may have been uncertain as to which of two images dominated when two were reported. Responses in our psychophysical experiments that were bimodally distributed (e.g., dashed lines in Fig 2E and 2F), for instance, resulted in mean angles (Fig 4A–4H) that were close to 0°. Small angles corresponding to such bimodal distributions were, in turn, interpreted as evidence that localization dominance was weak or did not occur. Thus:

uncertainty = (2 –P) / 2 (9)

where *P* is the proportion of trials in which subjects were predicted to indicate an additional image (Fig 13I–13L). The term is limited to values above 0.5 because some subjects continued to experience localization dominance even when the alone segments were long and fusion was weak (Fig 4B, 4C, 4F and 4G).

The last term, *symmetry*, assumes that localization dominance should not occur if the images that subjects perceive are symmetric around the midline:

$$symmetry=-1+(2/1+e^{-(dom_{right}+dom_{left})/0.025})$$ (10)

where *dom*_*left*_ and *dom*_*right*_ are measures of dominance obtained, respectively, for the left and right ICs by Eqs 8 and 9. This term differs from the previous two terms (Eqs 8 and 9) in that it does not directly involve fusion (*P*). The term’s greatest effect is when an intermediate level of fusion was predicted (*P* ≈ 0.5, Fig 13I–13L). Symmetry values approached or equaled zero, for instance, when the stimuli were independent and there was no lead-lag delay (leftmost points, blue lines, Fig 14J–14O) or when the stimuli were independent and the alone segments were excised (blue lines, Fig 14P and 14Q).

In most major respects, predictions obtained in this way (Fig 14J–14Q) resemble our behavioral results if we assume that *LI* (Fig 14B–14I) and dominance (Fig 14J–14Q) values of ~0.5 correspond to a reported angle of approximately 45 degrees (Fig 4A–4H), i.e., if we assume that indications on the arcs (Fig 1C) averaged ~45° when ~75% of half-maximal activities corresponded to positive (or negative) ITD. When the noise-pairs were identical, for instance, both our predictions and behavioral results are highly positive for delays shorter than ~4 ms, as is evident by comparing the filled circles and solid lines in Fig 4A–4H with those in Fig 14J–14Q. Both values also generally increased for independent noise-pairs having longer alone segments (i.e., longer lead-lag delays; open circles and dashed lines in Figs 4A–4C, 4E–4G and 14J–14O).

Contributions to localization dominance from the alone and superposed segments may also be inferred. Contributions from the alone segments, for instance, may be deduced from the values obtained with independent noises (open circles in Fig 14J–14Q), whereas contributions from the superposed segment may be deduced by comparing values obtained for the identical (filled circles, red lines) and independent noises (open circles, blue lines).

### Empirical predictions and signal-to-noise ratios

One aspect of our behavioral results that is only partially accounted for by our empirical predictions is the effect of the superposed segment’s length, especially on the independence ratio, obtained when the noise-pairs were independent and there was no delay (0 ms). An additional image was nearly always reported when this stimulus was long (200 ms; leftmost open circles in Fig 3A), for example, but was rarely reported when the stimulus was short (10 ms; leftmost open circles in Fig 3C). Our empirical measures also do not consider that reports on the arcs for the longer stimuli were bimodal (open circles in Fig 2A and 2B) but reports for the shorter stimuli were widely scattered and unimodal (open circles in Fig 2M and 2N). Thus, how ITDs were distributed *across trials*, when gauged by subjects’ reports on the arcs, provides little indication as to why additional images were reported.

**Fig 15:**
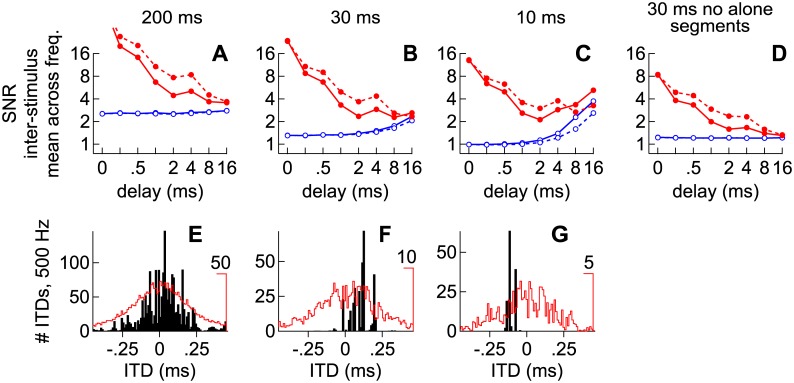
Signal-to-noise ratios of half-maximal activity patterns. (**A-D**) Signal-to-noise ratios (SNR) when noise-pairs were (A) 200 ms, (B) 30 ms, (C) 10 ms, or (D) 30 ms but lacking alone segments. Distributions of ITD (black bars), for the 500 Hz band, when a single pair of independent noises was superposed for (**E**) 200 ms, (**F**) 30 ms, or (**G**) 10 ms. Red lines show how ITDs were distributed, on average, across 100 different noise-pairs.

Signal-to-noise ratios (SNR), computed as the ratio of half-maximal activities, summed across frequency and ITD, over the sums of their standard deviations (Fig 15A–15D), provide a possible explanation. As previously described (see Figs 11I–11P and 12I–12P), a high SNR indicates that the pattern of activity obtained on any single trial was likely to resemble the pattern obtained, on average, across trials (i.e., for individual noise-pairs). In contrast, a low SNR indicates that the patterns were likely to vary from trial-to-trial.

To appreciate why these SNR values may have influenced when additional images were reported, it is necessary to first consider that ITD measurements were often clustered during individual carrier cycles (e.g., Fig 5D). Which, as an aside, is why ITD measurements obtained from zero-crossings, and thus from cycle-to-cycle, can be accumulated into frequency-specific distributions of ITDs similar to those obtained in the current study (not shown). As illustrated for the 500 Hz band in Fig 15E–15G, this clustering means that distributions of ITDs could be quite narrow when ITDs were accumulated for only a handful of carrier cycles (e.g., ~10 ms or 5 cycles at 500 Hz; Fig 15G) or rather broad when accumulated for as long as 200 ms (~100 cycles; Fig 15E). Indeed, frequency-specific distributions of ITDs for the 10 ms condition (Fig 15G) were often as narrow as when the noise-pairs were identical, which may explain why reports of additional images were infrequent under both conditions. For comparison, red lines in Fig 15E–15G show how ITDs were distributed, on average, across 100 different noise-pairs.

## Discussion

In the present study, contributions to precedence phenomena from the alone and superposed segments of identical or independent noise-pairs of various lengths are estimated. A neural model is then considered to explain our behavioral results, in which spatial cues are weighted by the envelopes of the filtered stimuli [27, 36], accumulated over time, and represented by sub-maximal activities across spatially selective neurons [10–13]. The model suggests that subjects’ experiences may simply reflect how spatial cues are represented and that specialized mechanisms for representing interaural coherence or suppressing reflections need not be invoked.

One question that arises from our model is how reports of multiple auditory “events” or “images” should be interpreted. Our interpretation thus far, which is similar to that of previous studies (e.g., [22, 46]), is that subjects may perceive multiple auditory events comparable to those that would have been perceived had the leading or the lagging sources been presented alone. One difficulty with this reductionist interpretation, however, is that it fails to consider the extent to which ITD (and ILD) can fluctuate when sounds from multiple sources or reflective surfaces are temporally superposed. We therefore offer that reports on both the upper and lower arcs (Fig 1C and 1D), under our experimental paradigm, may have reflected the left and right edges of a single auditory “object,” as opposed to the centroids of spatially distinct auditory images.

### Additional or alternative spatial representations

To describe the “edge” model, comparisons are drawn with a more conventional “maximum” model, in which spatial perception is viewed as corresponding to activity maxima (Fig 9C–9E) and thus the peaks of spatial cue distributions (e.g., Fig 8). The maximum model provides little indication as to why subjects would report additional images, when spatial cues are broadly distributed across low frequency bands, and is therefore at least partially rejected. Yet, under conditions when sub-maximal activities may represent the edges of spatial cue distributions, and possibly the edges of auditory objects, maximal activities may indicate their centers of mass. In this way, the peaks of spatial cue distributions and activity maxima may still provide important spatial information under the edge model.

Without invoking additional unexplored mechanisms, the inter-hemispheric hypothesis also provides little indication as to why subjects would report additional images when spatial cues are broadly distributed across low frequency bands [1, 4, 5]. Moreover, support for this hypothesis lies mainly in the argument that neurophysiological findings in mammals are incongruent with the maximum model under a narrow range of experimental conditions, when fluctuations in spatial cues are small [3–5].

In contrast to the maximum and inter-hemispheric hypotheses, neurophysiological findings in mammals [3–5] and owls [47, 48] are not only consistent with the edge model but required. The edge model, for instance, requires neurons with best ITDs that, in some animals, may extend well beyond their “physiological ranges” (e.g., [3, 4]). This requirement is evident in Fig 10N–10Q, where half-maximal activities near ± ~0.125 cycles (green circles in Fig 10O and 10P) would not have been obtained had neurons with best IPDs as large as ± ~0.25 cycles not been considered by our model. The significance of this is that a best IPD of 0.25 cycles corresponds to an ITD of 0.5 ms, at 500 Hz, which is several times larger than the “physiological range” of an animal like the gerbil (±0.135, [4, 49]). In other words, the best IPDs of neurons may often greatly exceed those that are generated by an animal’s head-related transfer function (HRTF) so that their slopes can represent the edges of spatial cue distributions under naturalistic conditions (e.g., [50]).

In addition to requiring at least some neurons with best IPDs as large as ± ~0.25 cycles, ambiguous half-maxima may be avoided, under the edge model, if best IPDs are also limited to values between approximately 0 and ± ~0.25 cycles (i.e., the π-limit) (e.g., [3, 4]). Ambiguous half-maxima corresponding to IPDs beneath the cross-hatchings in Fig 10H, near ±0.3 cycles, for instance, may contribute less to spatial perception if there are few or no neurons with best IPDs beyond ± ~0.25 cycles. Neurons with best IPDs between approximately 0 and ± ~0.25 cycles (Fig 9C) are also consistent with the edge model in the sense that their medial slopes and half-maxima (Fig 9F) span the midline [3, 4, 13] where spatial acuity is best in humans [51]. Thus, in our view, the best IPDs of laboratory mammals [3, 4] provide considerable support for edge model, as opposed to evidence against a narrow interpretation of the maximum model.

### Effects of frequency

High frequency bands (> 1.5 kHz) were excluded from our model because results from previous psychophysical studies [26, 35, 44, 45], in addition to results from our own model, suggest that contributions to human precedence phenomena may weaken as frequency increases. In Fig 10Q, for example, listeners are predicted to perceive two edges in directions corresponding to IPDs near ±0.125 cycles (green circles), which should correspond to substantially larger ITDs in lower frequency bands (e.g., ITDs ≈ ±0.125, ±0.25, and ±0.5 ms, respectively, at 1, 0.5 and 0.25 kHz). The envelopes of frequency bands above ~1.5 kHz may also generate significant energy above ~150 Hz (Fig 6D) and thus either lack representation [41] or cause dissimilar envelope features to overlap, as opposed to similar features [27, 36].

Another reason for excluding high frequency bands (> 1.5 kHz) is because representations derived from our model are averaged across frequency to simplify presentation (0.2–1.5 kHz; Figs 11 and 12). The strongest effects of precedence, across low frequency bands, are therefore already obscured and would have been further obscured had higher frequencies been incorporated. Indeed, our modeled results were averaged across frequency mainly to simplify presentation and because subjects in our behavioral experiments were not asked to provide separate reports for different frequency bands.

Lastly, while high frequencies are neither expected [26] nor predicted to contribute to localization dominance, except for when the lead-lag delay is very short, envelopes generated by high frequency filters may still contribute substantially to reports of additional images (i.e., fission). Neurons selective for envelope ITDs [52, 53], for instance, may generate distributions of ITDs that are just as broad as those by generated by neurons responsive to low frequency carriers. This could explain why additional images have been reported when independent noise-pairs are high-pass filtered [46].

### Effects of time, envelopes, and spatial cue distributions

Investigations of temporally superposed or binaurally independent stimuli have typically used noise-pairs that are quite long (e.g., 150–500 ms, [26, 46, 54]) or practically continuous (e.g., [6, 25]), suggesting that spatial cues may be “integrated” for considerable lengths of time. Our results further support this interpretation in that “independence proportions” increased considerably as our noise-pairs were lengthened (horizontal lines in Fig 3A–3C). For this reason, subjects’ perceptions and behavioral responses were modeled after weighting and accumulating spatial cues over the entire lengths of our stimuli.

Because our model attributes weights to spatial cues based on frequency-specific fluctuations in amplitude (Figs 5 and 6), it could be argued that our model also integrates spatial cues on much finer time-scale. The envelope of a noise filtered at 500 Hz, for instance, contains significant energy up to ~50 Hz (black line in Fig 6D). As a result, weights are attributed across the peaks and troughs of envelopes at a similar rate (50 Hz) and thus with an average period of approximately 20 ms (50 Hz^-1^ = 20 ms; see Fig 6A). One result of this envelope weighting is that cues near the onsets of our stimuli were almost always heavily weighted, since this was the only time when the envelopes of the filtered noise-pairs rose in unison. When averaged across a broad enough range of frequencies, weights following a transient even resemble the onset sensitivity functions of previous studies [55, 56]. In contrast, envelopes generally declined and were lightly weighted near the offsets of the noise-pairs.

Despite our use of envelope weights, one might still argue that the use of a single “time-window” is unreasonable since the lead and lag-alone segments of our longer, 200-ms, noise-pairs may have caused subjects to perceive two or more *temporally* distinct auditory events. We do not exclude this possibility, although how these events may have contributed to our behavioral results is unclear. When the lead and lag-alone segments were as long as 8 or 16 ms, for instance, reports of additional images could not have become much more frequent (Fig 3E) and their contributions to localization dominance were small (Fig 4I).

Because spatial cues were weighted and accumulated over the entire lengths of our stimuli, distributions of ITD were often bimodal, as opposed to unimodal, especially when the lead and lag-alone alone segments of our experimental stimuli were long. Even without these alone segments, the distributions were sometimes bimodal within some frequency bands (e.g., Fig 7A). These differences in modality, however, appear to be of little importance in the present study. Empirical measures of fusion and localization dominance obtained for stimuli whose ITD distributions were almost entirely unimodal, for instance, were similar to those obtained when the distributions were strongly bimodal (e.g., independent, 200 ms, noise-pairs versus identical, 10 ms, noise-pairs with 16 ms delay). One reason for these results is that distinct modes, which are closely spaced (e.g., separated by < ~0.25 cycles), may no longer be distinct when represented by neurons whose selectivity for ITD is broad across low frequency bands (Fig 9A). Empirical measures of fusion and localization dominance may also simply fail to detect differences in perception arising from distributions that are unimodal or multimodal in a given frequency band.

### Implications for other experimental and naturalistic sounds

Fluctuations in spatial cues are typically inferred by cross-correlating the signals in a listener’s ears. How spatially selective neurons may respond to a given stimulus, and how the stimulus may be perceived, is then inferred from the correlation (ρ). One such inference, suggested by the “maximum” model, is that a high correlation may evoke a narrow pattern of activity across a small number of similarly “tuned” neurons, whereas a low correlation may evoke a broad pattern across many different neurons. This inference is attractive in that auditory “images” could possibly resemble, or even mirror, how spatial cues are distributed (e.g., Figs 7 and 8). On the other hand, humans do not perceive a single intracranial event that broadens as interaural correlation is lowered but seem to perceive an auditory event that “splits,” so that one event is perceived in the left ear and an additional event is perceived in the right ear [6, 9]. We suggest that subjects in our experiments may have experienced a similar “splitting” effect when spatial cues were broadly distributed and that the edge model provides a possible explanation.

The edge model may also explain why humans are highly sensitive to *differences* in correlation (Δρ) [7, 8]. Specifically, it may be that listeners can readily detect when maximal and sub-maximal activities correspond to slightly different ITDs or that listeners can detect when sub-maximal activities in the left and right hemispheres correspond to slightly different ITDs. In other words, listeners may readily detect when auditory objects acquire edges and detect when existing edges expand or contract, as may occur under most naturalistic listening conditions.

## Supporting Information

S1 Fig Procedure for attributing cues to the superposed and alone segments. In attributing cues to the lead-alone, superposed, and lag-alone segments, it is necessary to incorporate the ringing of, frequency-specific, peripheral filters [28–31]. The impulse shown in panel **A**, for instance, has a center frequency at 500 Hz and its length is almost 25 ms. Convolution with such a filter blurs the distinction between each segment. Cues attributed to the lead-alone segment were therefore measured from the beginning of the stimulus, for the length of the lead-lag delay (4 ms), plus the time it took each frequency-specific impulse to reach its maximum amplitude (panel **B**). Cues attributed to the superposed segment were measured from the end of the lead-alone segment but within a time-frame equal to the length of the unfiltered superposed segment. Cues were attributed to the lag-alone segment if they were measured after the end of the superposed segment, provided that the filtered stimulus’ amplitude envelope remained above 20 dB (re: unfiltered noise bursts of 70 dB, RMS, re: 20 μPa). Importantly, cross hatching in panel **B** shows how the filtered leading and lagging stimuli may remain partially overlapped during the “alone” segments (4 ms), and especially during the lag-alone segment since the leading stimulus may ring for several milliseconds after its offset. At shorter delays, the filtered leading and lagging stimuli may also be substantially overlapped during the lead-alone segment, especially in lower frequency bands (delay < ~frequency^-1^).
(PDF)
