# Supplementary material for: A Neural Model of Auditory Space Compatible with Human Perception under Simulated Echoic Conditions
Source: PLoS One. 2015 Sep 10;10(9):e0137900. doi: 10.1371/journal.pone.0137900 (PMC4565656; doi:10.1371/journal.pone.0137900)
Supplement: S1 Fig — In attributing cues to the lead-alone, superposed, and lag-alone segments, it is necessary to incorporate the ringing of, frequency-specific, peripheral filters [28–31]. The impulse shown in panel A, for instance, has a center frequency at 500 Hz and its length is almost 25 ms. Convolution with such a filter blurs the distinction between each segment. Cues attributed to the lead-alone segment were therefore measured from the beginning of the stimulus, for the length of the lead-lag delay (4 ms), plus the time it took each frequency-specific impulse to reach its maximum amplitude (panel B). Cues attributed to the superposed segment were measured from the end of the lead-alone segment but within a time-frame equal to the length of the unfiltered superposed segment. Cues were attributed to the lag-alone segment if they were measured after the end of the superposed segment, provided that the filtered stimulus’ amplitude envelope remained above 20 dB (re: unfiltered noise bursts of 70 dB, RMS, re: 20 μPa). Importantly, cross hatching in panel B shows how the filtered leading and lagging stimuli may remain partially overlapped during the “alone” segments (4 ms), and especially during the lag-alone segment since the leading stimulus may ring for several milliseconds after its offset. At shorter delays, the filtered leading and lagging stimuli may also be substantially overlapped during the lead-alone segment, especially in lower frequency bands (delay < ~frequency-1). (PDF) [file pone.0137900.s001.pdf]

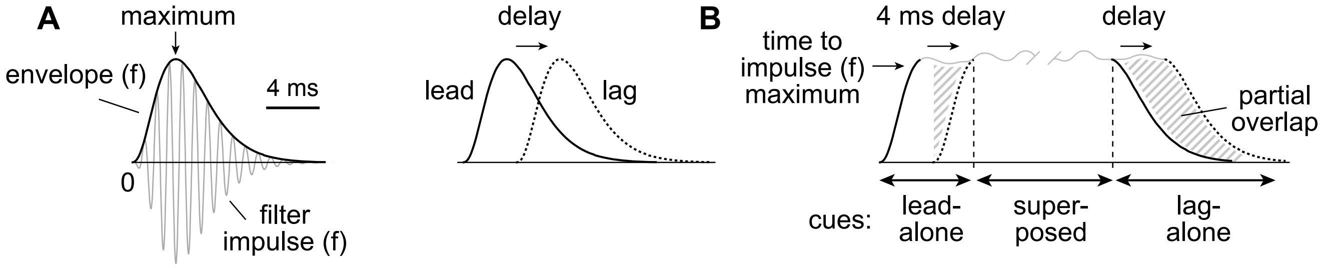

S1 Fig. Procedure for attributing cues to the superposed and alone segments

In attributing cues to the lead-alone, superposed, and lag-alone segments, it is necessary to incorporate the ringing of, frequency-specific, peripheral filters [28-31]. The impulse shown in panel **A**, for instance, has a center frequency at 500 Hz and its length is almost 25 ms. Convolution with such a filter blurs the distinction between each segment. Cues attributed to the lead-alone segment were therefore measured from the beginning of the stimulus, for the length of the lead-lag delay (4 ms), plus the time it took each frequency-specific impulse to reach its maximum amplitude (panel **B**). Cues attributed to the superposed segment were measured from the end of the lead-alone segment but within a time-frame equal to the length of the unfiltered superposed segment. Cues were attributed to the lag-alone segment if they were measured after the end of the superposed segment, provided that the filtered stimulus' amplitude envelope remained above 20 dB (re: unfiltered noise bursts of 70 dB, RMS, re: 20 μPa). Importantly, cross hatching in panel **B** shows how the filtered leading and lagging stimuli may remain partially overlapped during the "alone" segments (4 ms), and especially during the lag-alone segment since the leading stimulus may ring for several milliseconds after its offset. At shorter delays, the filtered leading and lagging stimuli may also be substantially overlapped during the lead-alone segment, especially in lower frequency bands (delay < ~frequency-1).
